# A network analysis on biopsychosocial factors and pain-related outcomes assessed during a COVID-19 lockdown

**DOI:** 10.1038/s41598-023-31054-4

**Published:** 2023-03-16

**Authors:** Carlos Gevers-Montoro, Bernard X. W. Liew, Zoha Deldar, Francisco Miguel Conesa-Buendia, Arantxa Ortega-De Mues, Deborah Falla, Ali Khatibi

**Affiliations:** 1 Madrid College of Chiropractic – RCU María Cristina, Paseo de los Alamillos 2, 28200 San Lorenzo de El Escorial, Madrid Spain; 2 Psychology Department, McGill University, Montréal, QC Canada; 3 School of Sport, Rehabilitation and Exercise Sciences, University of Essex, Colchester, Essex UK; 4 Bone and Joint Research Unit, Institute of Health Research (IIS–Fundación Jiménez Díaz Hospital), Madrid, Spain; 5 Centre of Precision Rehabilitation for Spinal Pain, School of Sport, Exercise and Rehabilitation Sciences, University of Birmingham, Birmingham, UK; 6 Centre for Human Brain Health, University of Birmingham, Birmingham, UK

**Keywords:** Pain, Anxiety, Musculoskeletal system

## Abstract

Psychological stress, social isolation, physical inactivity, and reduced access to care during lockdowns throughout a pandemic negatively impact pain and function. In the context of the first COVID-19 lockdown in Spain, we aimed to investigate how different biopsychosocial factors influence chiropractic patients’ pain-related outcomes and vice-versa. A total of 648 chiropractic patients completed online questionnaires including variables from the following categories: demographics, pain outcomes, pain beliefs, impact of the COVID-19 pandemic, stress/anxiety and self-efficacy. Twenty-eight variables were considered in a cross-sectional network analysis to examine bidirectional associations between biopsychosocial factors and pain outcomes. Subgroup analyses were conducted to estimate differences according to gender and symptom duration. The greatest associations were observed between pain duration and pain evolution during lockdown. Participants’ age, pain symptoms’ evolution during lockdown, and generalized anxiety were the variables with the strongest influence over the whole network. Negative emotions evoked by the pandemic were indirectly associated with pain outcomes, possibly via pain catastrophizing. The network structure of patients reporting acute pain showed important differences when compared to patients with chronic pain. These findings will contribute to identify which factors explain the deleterious effects of both the pandemic and the restrictions on patients living with pain.

## Introduction

A few weeks after confirmation of the first case of infection with SARS-CoV-2 in Spain^1^, the World Health Organization declared COVID-19 a pandemic^2^, and Spain went into full lockdown^3^. Spain experienced the highest number of cases in Europe during the first wave of the pandemic^1^. Two years later, the toll taken by the COVID-19 pandemic is undeniable, with over 6,000,000 deaths globally, 106,000 in Spain alone^4^. Beyond the direct impact on morbidity and mortality, the mitigation strategies carry an additional socio-economic impact of unfathomable dimensions^5^.

Mounting evidence is unveiling deleterious effects of the strict restrictions on mental and physical health^6–8^. In the general population, increasing symptoms of depression, anxiety and stress were reported^9^. Social distancing mandates came at the high cost of increasing social isolation and loneliness, often for the most vulnerable^10,11^. Some workers were pushed to unemployment while the economy shrank^12^. Financial strain and fear of illness both contributed to increasing levels of stress and uncertainty^6,8,13^. Pandemic-related intolerance of uncertainty was shown to be associated with psychological symptoms, particularly anxiety^14,15^, the most frequently reported throughout the pandemic in Spain^16,17^.

Psychological stress, combined with social isolation, and shrinking levels of physical activity^18–20^ may negatively impact pain conditions^13,21–24^. A variety of services for pain management, including chiropractic, were not accessible during lockdown in Spain^25^. Lockdown measures were associated with increased perceived pain severity and interference, particularly for individuals with chronic pain^26–28^. Pain catastrophizing partially mediated worsening pain outcomes^21,29^, which were influenced by factors such as change in pain treatment^26,30,31^, decreased physical activity^21,27^, kinesiophobia^25,32^, employment status and gender^29,33^. Notably, this negative impact of the pandemic on pain and psychological symptoms may be moderated by higher levels of self-efficacy^15,34^. Considering the complexity and multidimensional nature of both pain and COVID-19, the interaction of these demographic, psychosocial, and pain-related factors needs further examination.

Network analysis has been used substantially to investigate complex bidirectional interactions in psychopathology^35–37^, including patients with COVID-19^38^, though only recently was it applied to pain research^39–41^. Statistically, the association between two variables calculated in network analysis is analogous to the beta coefficient in a traditional multiple linear regression model^42^, while simultaneously analysing how each variable is related and adjusted to all other variables in the model. For example, poor sleep quality is associated with greater pain experience but greater pain can result in poor sleep^43^, a reciprocal relationship that is better suited to modelling via network analysis. Given that there is insufficient knowledge about the intricate relationships between biopsychosocial variables influencing pain conditions, scarce prior knowledge on the consequences of the pandemic on these conditions, and, the plausibility that these variables could be reciprocally related, network analysis represents the most appropriate technique for exploring how the pandemic influenced individual factors and clinical presentations.

We aimed to investigate, via network analysis, how different biopsychosocial factors interact with chiropractic patients’ pain-related outcomes, in the context of the COVID-19 pandemic. Specifically, we aimed to identify the most important associations, and to examine whether these differ by gender, and among patients with acute and chronic pain presentations. We hypothesized that psychosocial variables linked to a negative impact of the pandemic would be associated with worsening pain outcomes, and that this impact would differ according to pain chronicity.

## Methods

### Ethical approval, study setting and design

This was an observational cross-sectional survey study, conducted using baseline data from a published pragmatic trial^25^. Ethical approval was obtained by the Madrid College of Chiropractic research ethics committee (reference 300420) and the study adhered to the principles of the Declaration of Helsinki. Participants provided informed consent prior to completing the questionnaire. Data was obtained online from patients of 51 private chiropractic clinics throughout the Spanish geography, from May 4 to May 11, 2020. This week was chosen for data collection, as it marked the end of the most stringent phase of lockdown in Spain^44^. During this period, the movements of Spanish citizens were severely restricted and non-emergency healthcare services, including chiropractic, were still not available.

### Patient recruitment

All clinics registered with the Spanish Chiropractic Association were invited to participate in the recruitment process via e-mail. A total of 51 chiropractic clinics representing 16 of the 17 autonomous regions in Spain expressed interest in participating in the study and recruiting patients. Individuals were eligible to participate if they were chiropractic patients residing in Spain before the government declared the state of emergency, reported acute or chronic pain at the time of the survey and were over 18 years of age. Participant clinics were instructed to generate a patient list and to randomly contact one of every three patients to participate in the study. This was done to reduce the odds of a sampling bias. Contacts were made via phone calls, e-mails, or text messages by the chiropractor or the administrative staff.

**Figure 1:**
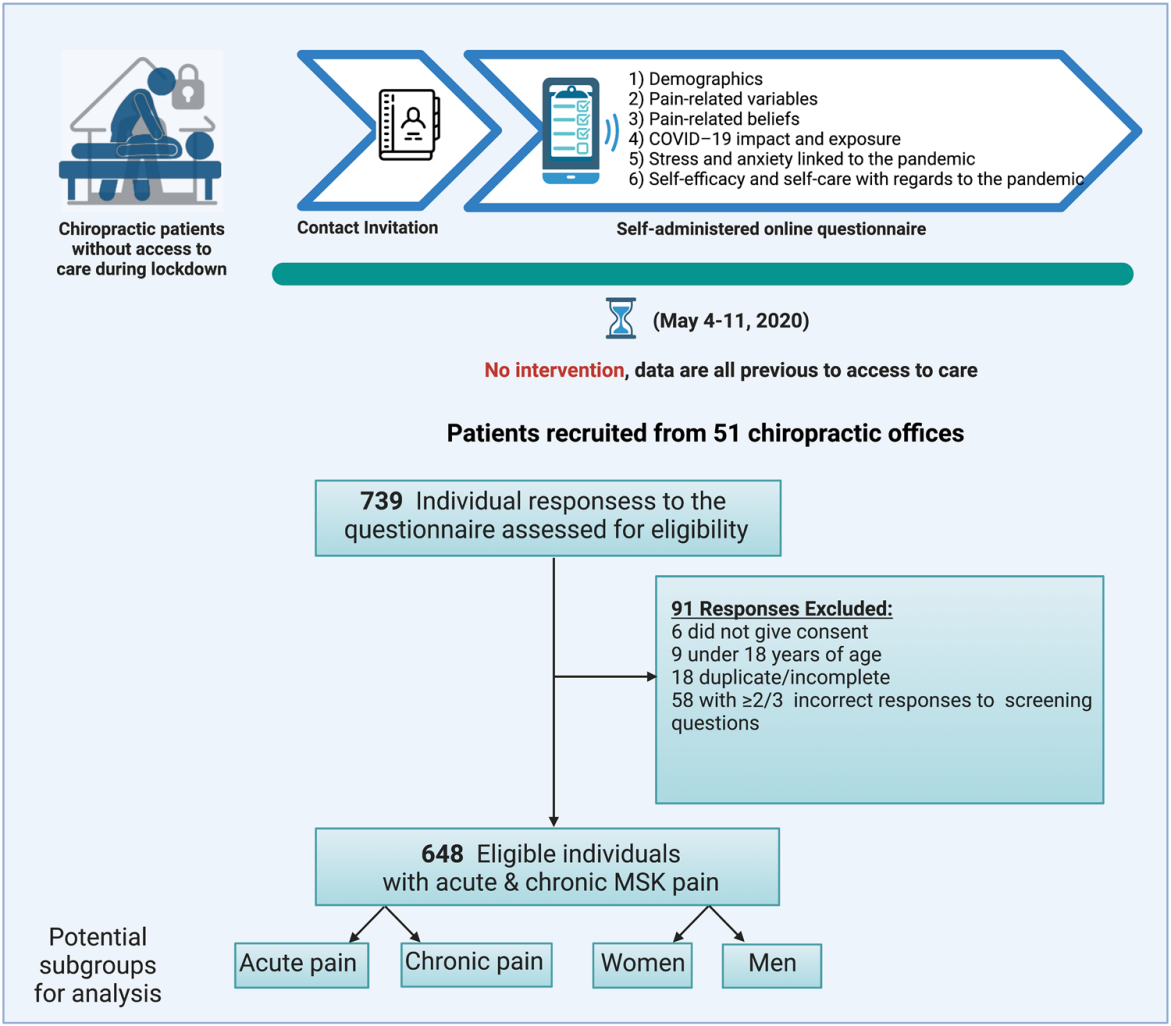
Data collection protocol. Representation of the process of data collection for the study, before implementation of the network analysis. Note: MSK = Musculoskeletal.

Patients accepting to participate were provided a link to access the online survey and were requested to complete it in the following 24 h. Access to the survey was secured for one week until May 11, when different regions in Spain were entering different phases of lockdown easing. A total of 739 responses were received and screened for duplicates, responses lacking consent and invalid responses, resulting in a final sample of 648 patients (see Fig. 1). Duplicates were considered when two or more identical answers to every question were found. Responses were considered non-valid for the study when participants answered incorrectly two of three attentional screening questions.

### Self-reported outcome measures included in the survey

The survey was designed and completed online using Google Forms (Google Inc., Mountain View, CA, US). The national chiropractic patient association (Asociación Española de Usuarios de Quiropráctica: http://www.aeuq.es/) was consulted on the format, design, length, and items included in the survey. The survey was pretested among patient partners and modified according to feedback provided by two patient representatives who participated in the creation of the questionnaires. Completing the survey required between 20 and 30 min. The survey comprised six separate sections with the following dimensions (see Fig. 1): demographics (first section), pain variables (second section), pain-related beliefs (third section), COVID-19 impact and exposure (fourth section), pandemic stress and general anxiety (fifth section), self-efficacy and self-care with regards to the pandemic (sixth and last section). Consent to participate was provided at the beginning of the first section. All items were written in Spanish, using the validated versions in Spanish of structured questionnaires when available.

#### First section: demographic variables

Participants were initially required to provide information on their age, gender identity, marital status, highest education level, region of residence, number of cohabitants, of these, whether one or more had been diagnosed with COVID-19, and finally on whether they suffer from any chronic comorbid condition (excluding chronic pain, which would be described in the next section) such as cancer, diabetes, cardiovascular, respiratory, rheumatological, autoimmune, musculoskeletal, endocrine or digestive chronic conditions.

#### Second section: pain variables

The second section comprised questions related to the patient’s current pain and pain-related beliefs. Initially, participants were asked about current pain features using ad-hoc items inquiring about the following: location(s), highest current pain intensity (numerical rating scale from 0 to 10, anchored at 0 = no pain at all, and 10 = maximum pain imaginable), pain duration (0 to 3 months, 3 to 12 months or more than 12 months) and frequency (constant, daily, weekly, occasionally), and a self-reported evolution of their pain since the beginning of lockdown (much improved, improved, no change, worsened, much worsened, new pain). Patients reporting that symptoms (for their main complaint) had started in the previous 0 to 3 months were categorized as acute, while patients reporting a symptom duration superior to 3 months were considered chronic. This classification is consistent with the International Association for the Study of Pain’s criteria for defining chronic pain^45^. Additionally, 6 items on the degree of pain interference were used from the Brief Pain Inventory (BPI)^46,47^. The interference scale in its validated version in Spanish has shown good reliability (Cronbach’s *ɑ* = 0.93)^46^. Considering that the confinement measures explicitly forbid walking outdoors, the patient representatives suggested excluding the item related to interference with “walking”, hence it was not used. This may impact the validity and reliability of this questionnaire, therefore, results involving pain interference must be interpreted with caution. Finally, participants were asked about any modifications of their pharmacological and non-pharmacological treatment during the lockdown, whether they would have continued visiting their chiropractor if this was an option, and on the length of time they had previously been under chiropractic care.

#### Third section: pain-related beliefs

Pain catastrophizing and kinesiophobia are cognitions that mediate pain responses and behaviours^48,49^. These multidimensional constructs were shown to influence pain intensity and interference^50^, also during the COVID-19 pandemic^25,29^. Pain catastrophizing targets rumination, magnification and helplessness^48^, while kinesiophobia usually encompasses the constructs of activity avoidance, somatic focus and fear of harm/re-injury^49^. In order to simplify the survey, we used the short 4-item version of the pain catastrophizing scale (PCS-4)^51,52^ and the short 11-item version of the Tampa Scale of Kinesiophobia (TSK-11)^53^. The PCS-4 has shown good internal consistency (*ɑ* = 0.86) and an almost perfect correlation with the long version (*r* = 0.96)^51^, which has been validated in Spanish^52^. The Spanish version of the TSK-11 has been validated and has shown good reliability and validity with a 2-factor solution, namely activity avoidance and harm^53^.

#### Fourth section: level of exposure and impact of the COVID-19 pandemic

In the fourth section, participants were asked whether they had symptoms of, had been diagnosed with, or had been in contact with someone diagnosed with COVID-19, and whether they had visited a healthcare professional in the previous two weeks, and if affirmative, what was the reason (pain, COVID-19, both or other) and which professional. The subsequent items inquired about what was the impact of the pandemic on participant’s employment status (employment before the pandemic, changes due to the pandemic, current status) and about which of the restrictions had affected their life the most. Finally, participants were asked to rate from 0 to 10 the degree to which they experienced nine different emotions when receiving information about the pandemic: sadness, worry, loneliness, anger, impotence, anxiety, surprise, relief, and hope.

#### Fifth section: stress linked to the COVID-19 pandemic and general anxiety

The fifth section consisted of seven questions regarding the degree of stress perceived with regards to the pandemic, the restrictions, one’s health, fear of economic difficulties, fear of food shortage, fear of resource shortage and loved one’s health, followed by the validated version in Spanish of the Generalized Anxiety Disorder scale (GAD-7)^54^, which has excellent reliability, and the short version of the Intolerance of Uncertainty Scale (IUS–12)^55^, which has not been validated into Spanish. The GAD-7 is a useful tool to self-report symptoms of anxiety^56^, frequently employed during the pandemic to assess these symptoms via online questionnaires^15,16^. Intolerance of uncertainty refers to the tendency to develop anxiety and avoid certain behaviours in face of the possibility of a negative event occurring^55^. This construct is considered instrumental in the aetiology and maintenance of anxiety^57^, particularly in situations with a high degree of uncertainty such as the COVID-19 pandemic^15,58^.

#### Sixth section: self-efficacy and self-care during the COVID-19 pandemic

The sixth and final section included the Spanish validated version of the General Self-Efficacy scale (SE-10)^59^. Self-efficacy refers to the self-belief on one’s abilities to cope during difficult tasks or in the face of adversity^60^. Lower levels are associated with depression, anxiety, stress, and symptoms, including pain^34,61^. Ten final items adapted from the Fear of Illness and Virus Evaluation scale (FIVE)^62^ were added to this section. The FIVE scale was developed by Dr. Ehrenreich-May to assess fears and behaviours potentially related to the pandemic^62^. Out of its 35 items, ten measuring avoidance and mitigation behaviours related to the virus were selected.

Embedded within the survey were three additional questions that served as instructional manipulation checks, attentional screening questions which have been shown to increase the reliability of the dataset^63^, resulting in a total of 105 items.

### Statistical analysis: approach to network analysis

#### Software and packages

The data set was analysed with the R software (version 4.0.0, available at https://www.r-project.org)^64^. Several packages were used to carry out the analyses, including *qgraph*^65^, and *mgm*^66^ for network estimation, and *bootnet*^67^ for stability analysis. All codes and results can be found on the public code hosting platform GitHub (https://bernard-liew.github.io/2020_ODI_network/).

#### Variables included in network analysis

A network structure is composed of nodes (variables influencing each other) and edges (connections or associations between nodes). In this analysis, individual factors are treated as *nodes*, and a network model reflects their relationships as a set of mutually interacting associations between these *nodes*. Associations between two nodes in a network are connected by an “edge” and reflect the magnitude of the relationship after statistically controlling for all other nodes in the network model^42^. In our study, the 28 variables in Table 1 were used as nodes and were included in the network model. These variables were selected as they were deemed to be more relevant to understand the relationship between psychosocial factors and pain-related outcomes during a COVID-19 lockdown. Edges represent the existence of an association between two nodes, conditioned on all other nodes. Each edge in the network represents either a positive regularized association (blue edges) or a negative regularized association (red edges). The thickness and colour saturation of an edge denotes its weight (the strength of the association between two nodes).

**Table 1:** Variables included in the network analysis.

Variables’ construct	Variables’ label	Description	Variable type	Levels*
Age	Age	Patient's age in years	Numeric	
Gender	Gender_^_	Patient self-identified gender (man, woman, other)	Categorical	**Man,** woman
Marital status	Marital_status	Marital status (single, married, divorced, widowed)	Categorical	**Married,** others
Level of education	Education	Level of education (none, basic, high school, university)	Categorical	**University,** others
Comorbidities	Comorbidities	Presence of chronic comorbid conditions	Categorical	**No,** yes
Number of pain sites	Number_pain_sites	Number of pain painful body regions reported	Numeric	
Pain intensity	Pain_intensity	Current pain intensity at site with highest pain	Numeric	
Pain duration	Chronicity*	Pain condition categorized as Chronic or Acute according to symptom duration (< or ≥ 3 months)	Categorical	**Acute,** chronic
Pain frequency	Frequency	Frequency of pain perceived (constant, daily, weekly, occasionally)	Categorical	**Constantly,** daily, weekly, occasionally
Pain evolution	Pain_evolution	Changes in pain symptoms since beginning of lockdown categorized as improve, no change, worse	Categorical	**Worse,** others
Changes in pain treatment	Change_in_tx	Did the patient modify his/her pain treatment during lockdown (Yes/No)	Categorical	**No,** yes
Pain interference	Interference	Pain interference from the Brief Pain Inventory (0–60)	Numeric	
Pain catastrophising	PCS	Pain Catastrophizing Scale, short version total (0–16)	Numeric	
Kinesiophobia	TSK	Tampa Scale of Kinesiophobia, short version, total (11–44)	Numeric	
Generalized anxiety	GAD	Generalized Anxiety Disorder scale, total (0–21)	Numeric	
Intolerance of uncertainty	IOUS	Intolerance of Uncertainty scale, total (12–60)	Numeric	
Self-efficacy	SE	General Self-Efficacy scale, total (10–100)	Numeric	
Employment status	Job	Employment status at the time of study	Categorical	**Full-time job,** others
COVID-19 sadness	Sad	The degree to which this emotion was evoked by the pandemic, on a scale from 0 to 10	Numeric	
COVID-19 worry	Worry	The degree to which this emotion was evoked by the pandemic, on a scale from 0 to 10	Numeric	
COVID-19 loneliness	Lonely	The degree to which this emotion was evoked by the pandemic, on a scale from 0 to 10	Numeric	
COVID-19 anger	Anger	The degree to which this emotion was evoked by the pandemic, on a scale from 0 to 10	Numeric	
COVID-19 helplessness	Helpless	The degree to which this emotion was evoked by the pandemic, on a scale from 0 to 10	Numeric	
COVID-19 anxiety	Anxiety	The degree to which this emotion was evoked by the pandemic, on a scale from 0 to 10	Numeric	
COVID-19 surprise	Surprise	The degree to which this emotion was evoked by the pandemic, on a scale from 0 to 10	Numeric	
COVID-19 relief	Relief	The degree to which this emotion was evoked by the pandemic, on a scale from 0 to 10	Numeric	
COVID-19 hope	Hope	The degree to which this emotion was evoked by the pandemic, on a scale from 0 to 10	Numeric	
COVID-19 stress	Stress_covid	Degree to which the patient finds the pandemic stressful, on a scale from 0 to 10	Numeric	

^^^Excluded from subgroup analysis between men and women.

*Excluded from subgroup analysis between acute and chronic.

Bold indicate reference level.

#### Network estimation

A Mixed Graphical Model was used to estimate the network^66^. Least absolute shrinkage and selection operator (LASSO) regularization was used during modelling to elicit a sparse model. Compared to a saturated model, a sparse model is one with a comparatively fewer number of edges to explain the covariation structure of the data—with the benefit that the ensuing model becomes more interpretable^42^.

#### Node centrality

Not all nodes in a network are equally important in determining the network structure^68^. Centrality indices provide a measure of a node’s importance, and they are based on the pattern of connectivity of a node of interest with its surrounding nodes—with the ensuing information potentially useful for guiding future interventions^69^. In the present study, we calculated the Strength Centrality measure for our networks. Strength Centrality is defined as the sum of the weights of the edges (in absolute value) incident to the node of interest^70,71^. Clinically, a high Strength node represents a logical and efficient therapeutic target, because a change in the value of this node has a strong direct and quick (because of its strong direct connections) influence on other nodes within the network.

#### Accuracy and stability

The accuracy of the edge weights was assessed using bootstrapping^67^. For this, the data is resampled with replacement, and a new set of edge weights and Centrality indices are calculated, which is repeated many times in this study. Herein, we used 1000 bootstrapped iterations, to generate 95% confidence intervals (CI) of all edge weights. These edge weight CIs reflect the uncertainty in estimated edge-weights and may be used to make a relative comparison of the different edge weights^67^. Given that the LASSO algorithm already retains only non-zero association edge, the presence or absence of an edge in the model should not be determined by the width of the CIs.

The stability of the calculated Centrality index was assessed using the case-dropping subset bootstrap^67^. This procedure drops a percentage of participants, recalculates the network and the Centrality Index. A Centrality-Stability coefficient (CS-coefficient) is then produced^67^. CS reflects the maximum proportion of participants that can be dropped, such that with 95% probability the correlation between the centrality value of the original and bootstrapped data would reach a certain threshold magnitude–current set at 0.7. It is suggested that CS_cor=0.7_ should not be below 0.25 and better if > 0.5^67^.

#### Subgroup analysis

Network estimation, node centrality, accuracy and stability analyses were conducted on the entire cohort (n = 648), with two subgroup analyses conducted—gender (women [n = 455] vs. men [n = 193]) and chronicity (acute [n = 201] vs. chronic [n = 447]).

## Results

**Figure 2:**
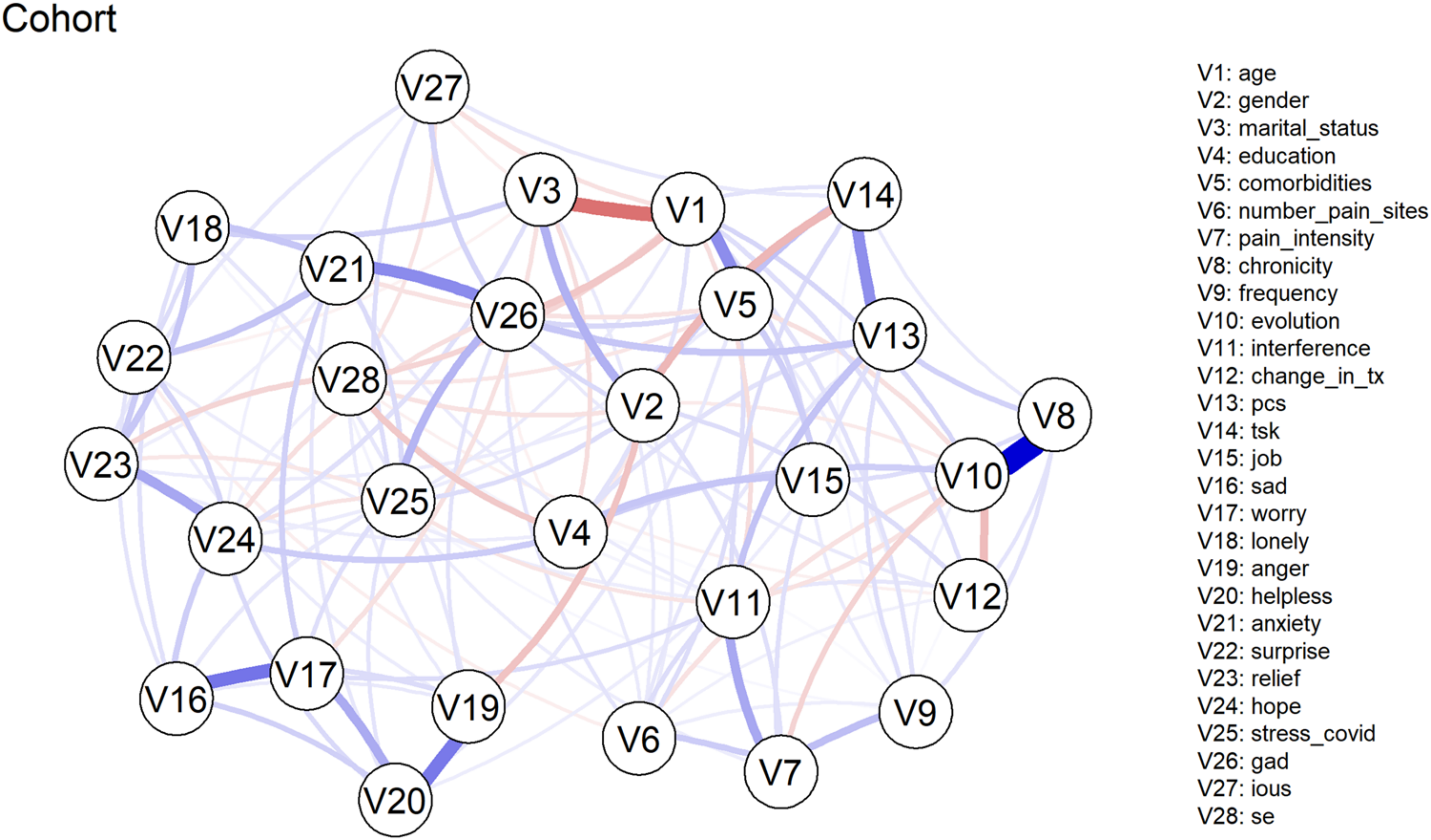
Network analysis of the entire cohort. Edges represent connections between two nodes and are interpreted as the existence of an association between two nodes, adjusted for all other nodes. Each edge in the network represents either positive regularized adjusted associations (blue edges) or negative regularized adjusted associations (red edges). The thickness and colour saturation of an edge denotes its weight (the strength of the association between two nodes).

**Figure 3:**
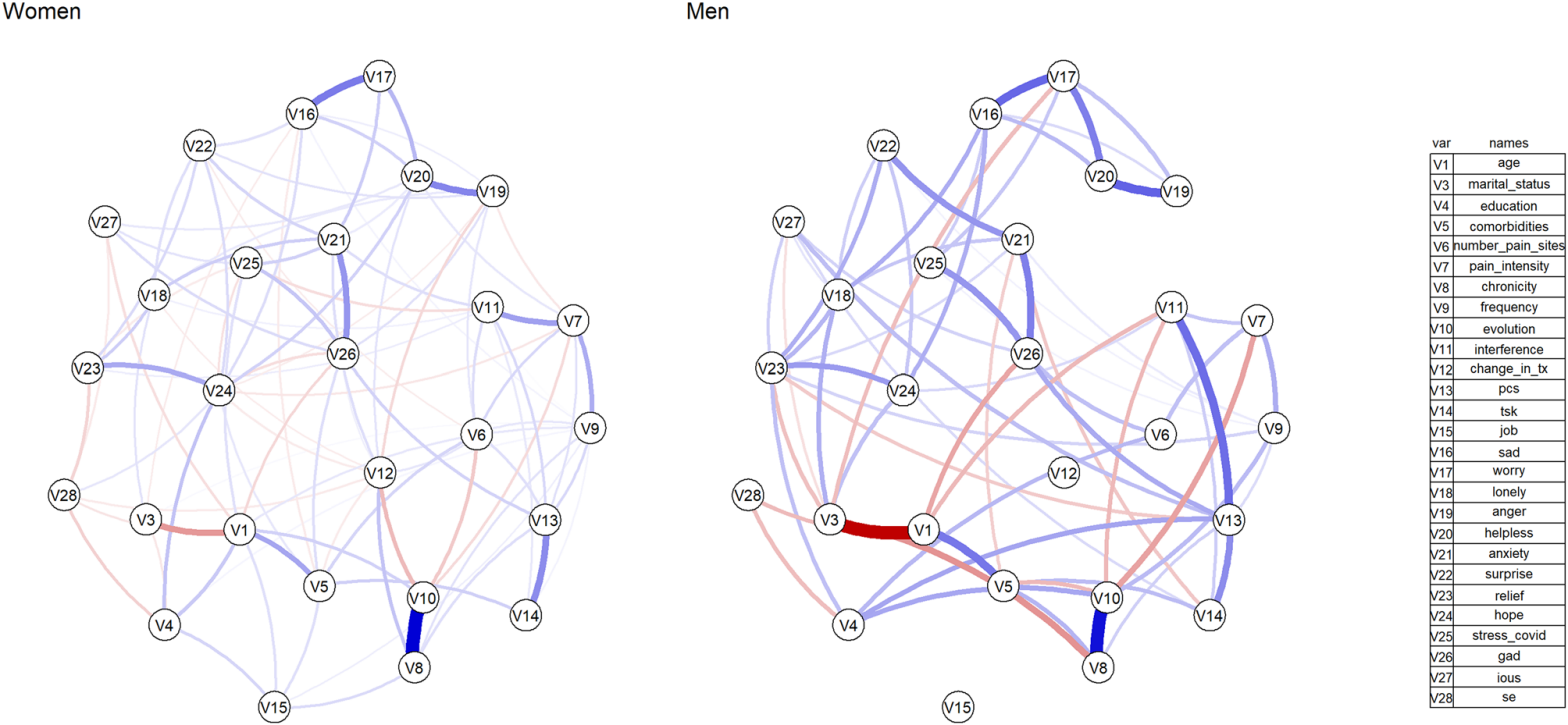
Subgroup network analysis for gender differences. Edges represent connections between two nodes and are interpreted as the existence of an association between two nodes, adjusted for all other nodes. Each edge in the network represents either positive regularized adjusted associations (blue edges) or negative regularized adjusted associations (red edges). The thickness and colour saturation of an edge denotes its weight (the strength of the association between two nodes).

**Figure 4:**
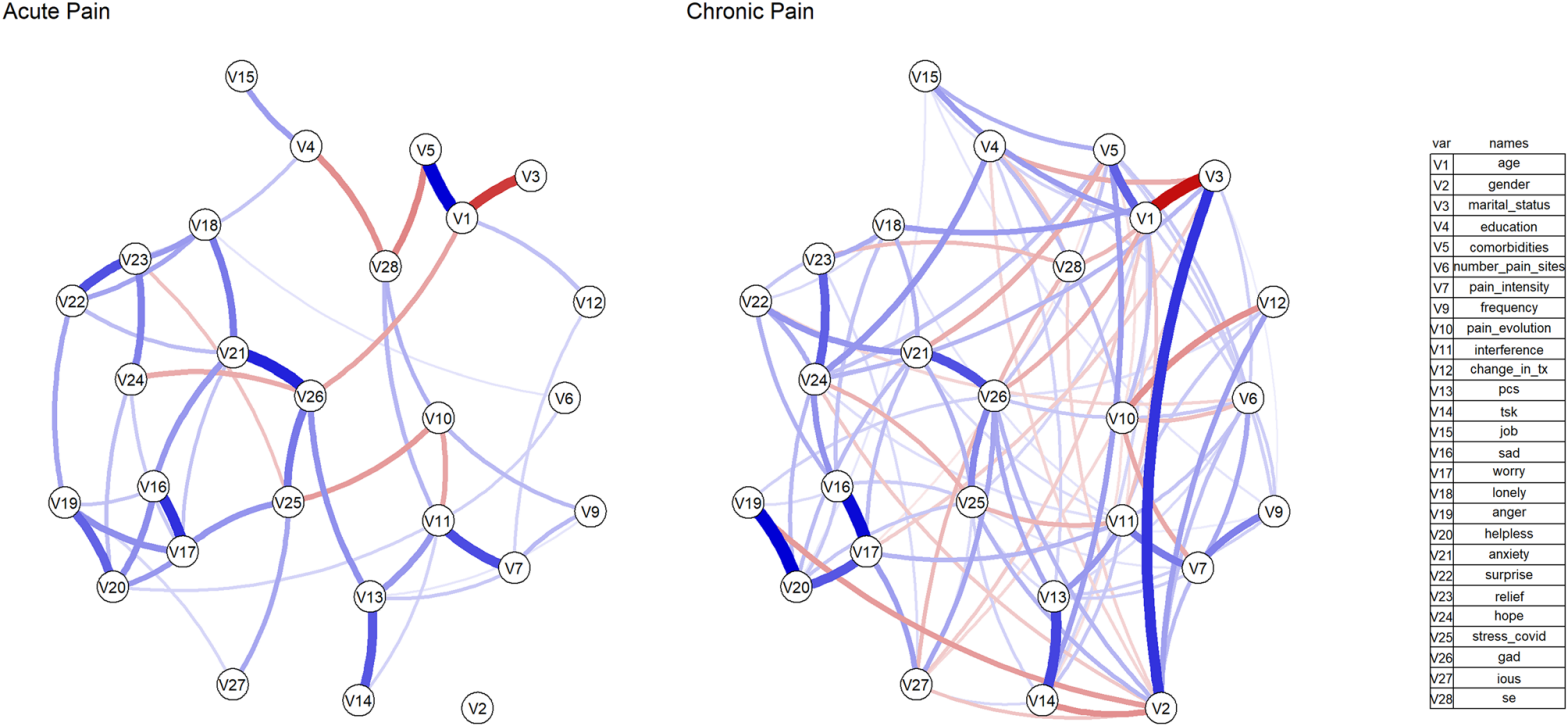
Subgroup network analysis for differences in pain chronicity. Edges represent connections between two nodes and are interpreted as the existence of an association between two nodes, adjusted for all other nodes. Each edge in the network represents either positive regularized adjusted associations (blue edges) or negative regularized adjusted associations (red edges). The thickness and colour saturation of an edge denotes its weight (the strength of the association between two nodes).

The descriptive characteristics of the variables (original scale) used in the network analysis can be found in Table 2. Figure 2 shows the network of the cohort-level analysis, Fig. 3 shows the networks of the first subgroup analysis for gender, and Fig. 4 shows the networks of the second subgroup analysis based on chronicity.

**Table 2:** Baseline descriptive characteristics of cohort.

Variables	Summary value
Age, mean (SD)	46.97 (12.51)
Gender, n (%)	
Women	455 (70.22)
Men	193 (29.78)
Marital status, n (%)	
Single	228 (35.19)
Married	356 (54.94)
Divorced	54 (8.33)
Widowed	10 (1.54)
Level of education, n (%)	
None	2 (0.31)
Primary	69 (10.65)
Secondary	189 (29.17)
University	388 (59.88)
Employment status, n (%)	
Full-time job	314 (48.46)
Part-time job	97 (14.97)
On sick leave	28 (4.32)
Unemployed	147 (22.69)
Retired	62 (9.57)
Region in Spain, n (%)	
Community of Madrid	163 (25.25)
Andalusia	132 (20.37)
Basque Country	126 (19.44)
Catalonia	78 (12.04)
Valencian community	32 (4.94)
Balearic Islands	26 (4.01)
Asturias	21 (3.24)
Extremadura	17 (2.62)
Other	41 (6.33)
Pain evolution, n (%)	
New pain	75 (11.57)
Much worse	93 (14.35)
Worse	236 (36.42)
No change	172 (26.54)
Improved	52 (8.02)
Much improved	20 (3.09)
Pain duration, n (%)	
< 3 months	201 (31.02)
3–12 months	86 (13.27)
> 12 months	361 (55.71)
Pain frequency, n (%)	
Constant	87 (13.43)
Daily	229 (35.34)
Weekly	96 (14.81)
Occasional	236 (36.42)
Pain treatment changed (yes), n (%)	442 (68.21)
Chronic comorbidities (yes), n (%)	158 (24.38)
Number of pain sites, mean (SD)	3.24 (2.06)
Pain intensity (0–10), mean (SD)	5.32 (2.16)
Pain interference (0–60), mean (SD)	17.95 (15.37)
Pain Catastrophizing Scale PCS-4 (0–16), mean (SD)	6.77 (3.28)
Tampa Scale of Kinesiophobia TSK-11 (11–44), mean (SD)	25.53 (5.54)
Generalized Anxiety Disorder scale GAD-7 (0–21), mean (SD)	5.45 (4.3)
Intolerance Of Uncertainty Scale IOUS-12 (12–60), mean (SD)	29.17 (10.19)
Self-Efficacy SE-10 (10–100), mean (SD)	73.62 (14.15)
COVID-specific emotions (0–10), mean (SD)	
Sadness	4.56 (3.13)
Worry	5.49 (3.06)
Loneliness	2.02 (2.6)
Anger	4.21 (3.3)
Helplessness	5.07 (3.39)
Anxiety	3.19 (3.1)
Surprise	2.56 (2.81)
Relief	1.7 (2.3)
Hope	4.34 (3.11)
COVID-specific stress (0–10), mean (SD)	7.05 (2.53)

### Edge weights and variability

**Figure 5:**
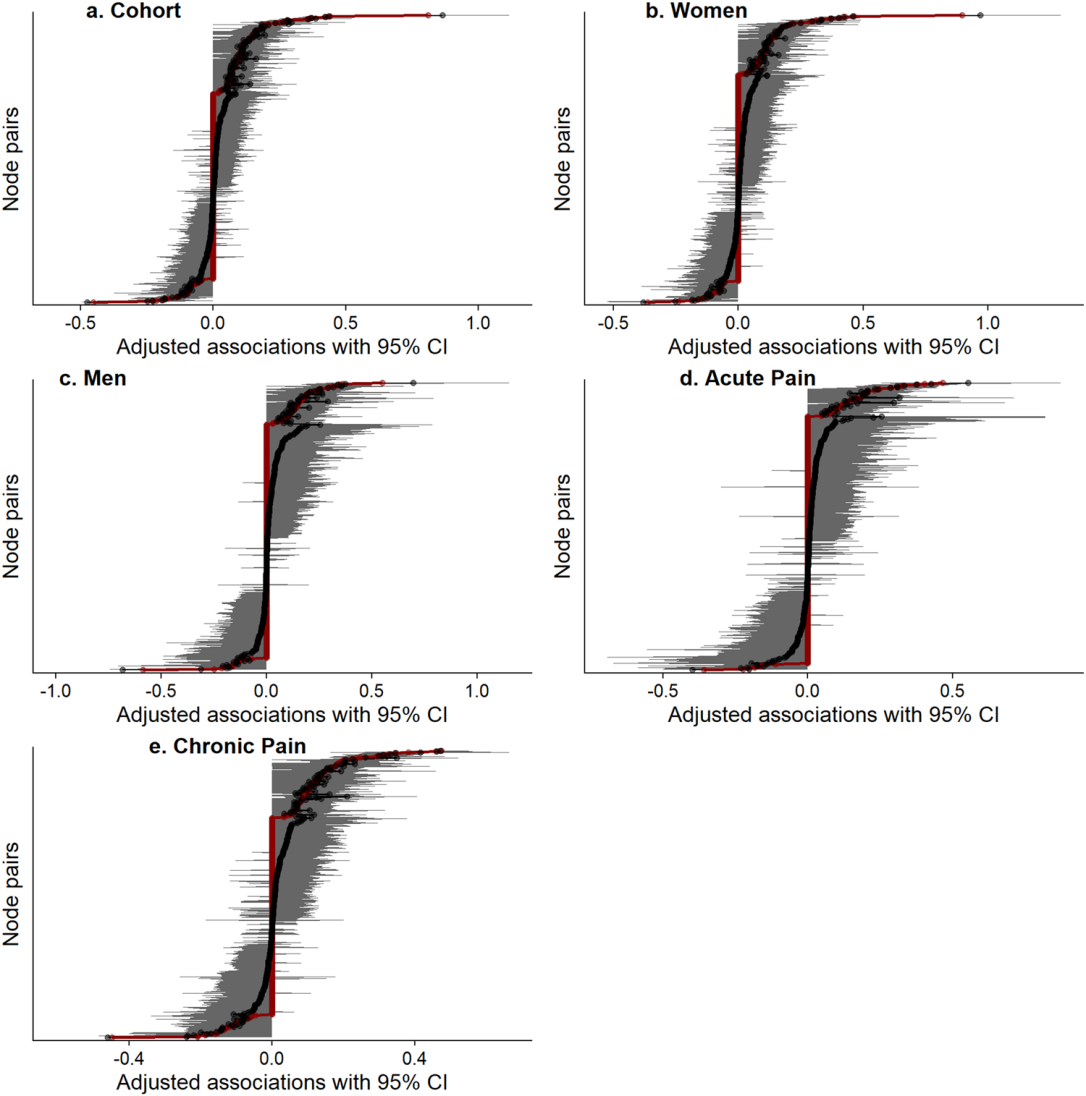
Estimated and observed mean of pairwise associations. A qualitative overview of the estimated (grey) and observed mean (red) with 95% CI of pairwise associations across different node pairs. The names of the node pairs (i.e. Y axis labels and ticks) are removed to avoid visual clutter. Exact values are reported in the Supplementary Material.

For the cohort analysis, the five greatest pair-wise association were between Chronicity-Pain Evolution (0.81 (95%CI [0.63 to 1.12]), Age-Marital status (− 0.45 (95%CI [− 0.59 to − 0.36])), Sadness and Worry (0.44 (95%CI [0.35 to 0.51])), Anger and Helplessness (0.42 (95%CI [0.34 to 0.50])), and COVID-specific and Generalized Anxiety (0.37 (95%CI [0.30 to 0.44])) (Figs. 2 and 5).

When the sample was divided based on gender, for women, the five greatest pair-wise association were between Chronicity-Pain Evolution (0.90 (95%CI [0.69 to 1.29]), Sadness and Worry (0.46 (95%CI [0.36 to 0.55])), Anger and Helplessness (0.43 (95%CI [0.32 to 0.52])), PCS-TSK (0.40 (95%CI [0.31 to 0.49])), and COVID-specific and Generalized Anxiety (0.37 (95%CI [0.29 to 0.46])) (Figs. 3 and 5). For men, the five greatest pair-wise association were between Age-Marital status (− 0.59 (95%CI [− 1.00 to − 0.41])), Chronicity-Pain Evolution (0.55 (95%CI [0.28 to 1.15]), Anger and Helplessness (0.36 (95%CI [0.19 to 0.53])), Sadness and Worry (0.35 (95%CI [0.17 to 0.50])), and Pain interference and PCS (0.34 (95%CI [0.21 to 0.50])) (Figs. 3 and 5).

The sample was divided in two subgroups according to their self-reported pain duration (acute vs. chronic). For individuals in acute pain, the five greatest pair-wise association were between Age-comorbidities (0.47 (95%CI [0.25 to 0.87]), COVID-specific and generalized Anxiety (0.40 (95%CI [0.32 to 0.53])), Sadness and Worry (0.38 (95%CI [0.22 to 0.52])), Age-Marital status (-0.36 (95%CI [− 0.62 to − 0.20])), and Pain intensity—Pain interference (0.33 (95%CI [0.20 to 0.45])) (Figs. 4 and 5). For individuals in chronic pain, the five greatest pair-wise association were between Anger and Helplessness (0.48 (95%CI [0.39 to 0.55]), COVID-specific and generalized Anxiety (0.46 (95%CI [0.36 to 0.56])), Age-Marital status (− 0.45 (95%CI [− 0.61 to − 0.32])), Gender-Marital status (0.38 (95%CI [− 0.16 to 0.66])), and PCS-TSK (0.35 (95%CI [0.25 to 0.43])) (Figs. 4 and 5).

### Centrality and variability

**Figure 6:**
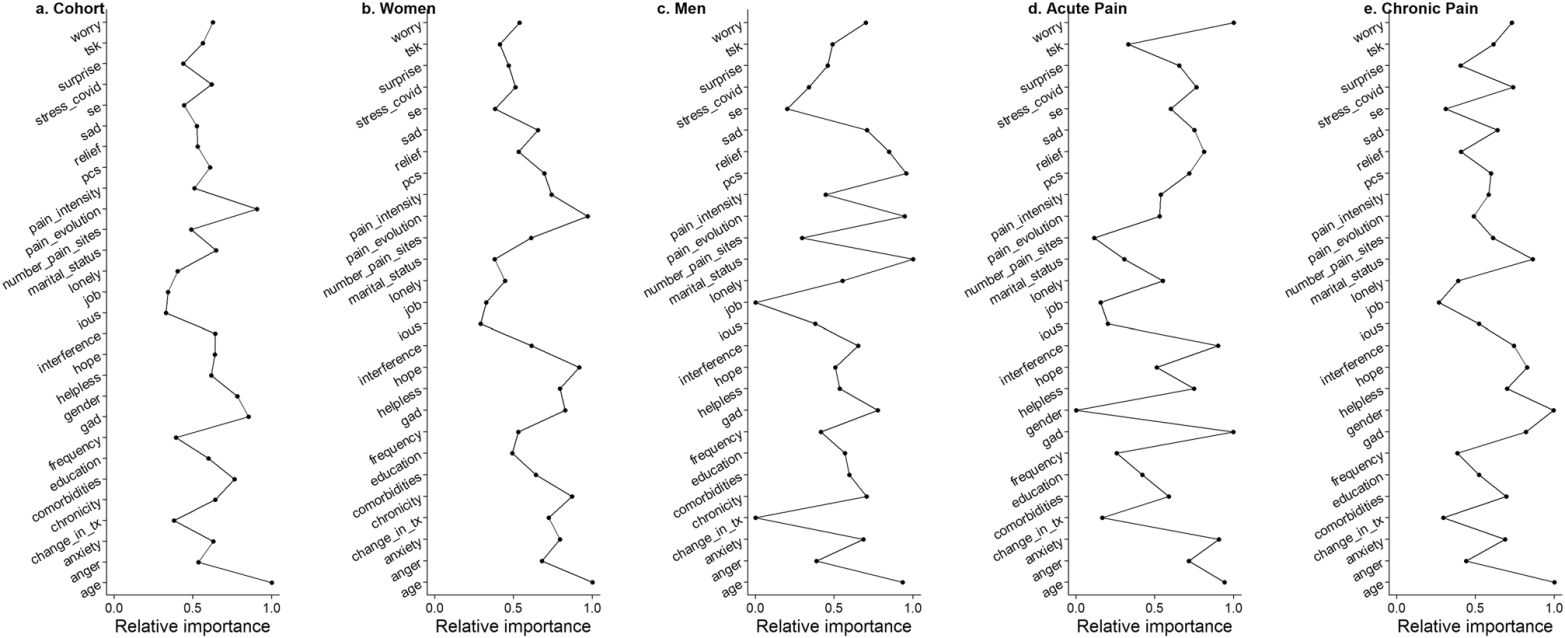
Centrality measures of Strength. Average correlations between centrality indices of networks sampled with persons dropped and networks built on the entire input dataset, at all follow-up time points.

For the cohort analysis, Age, Pain Evolution, and GAD were the nodes with the top three highest Strength values (Fig. 6). For the subgroup analysis of Gender, the most important three nodes were Age, Pain Evolution, and Hope for women, whilst for men, the three nodes were Marital status, PCS, and Pain evolution (Fig. 6). For the subgroup analysis of chronicity, the most important three nodes were Worry, GAD, and Age for acute pain sufferers, and for those in chronic pain, they were Age, Gender, and Marital Status (Fig. 6).

**Figure 7:**
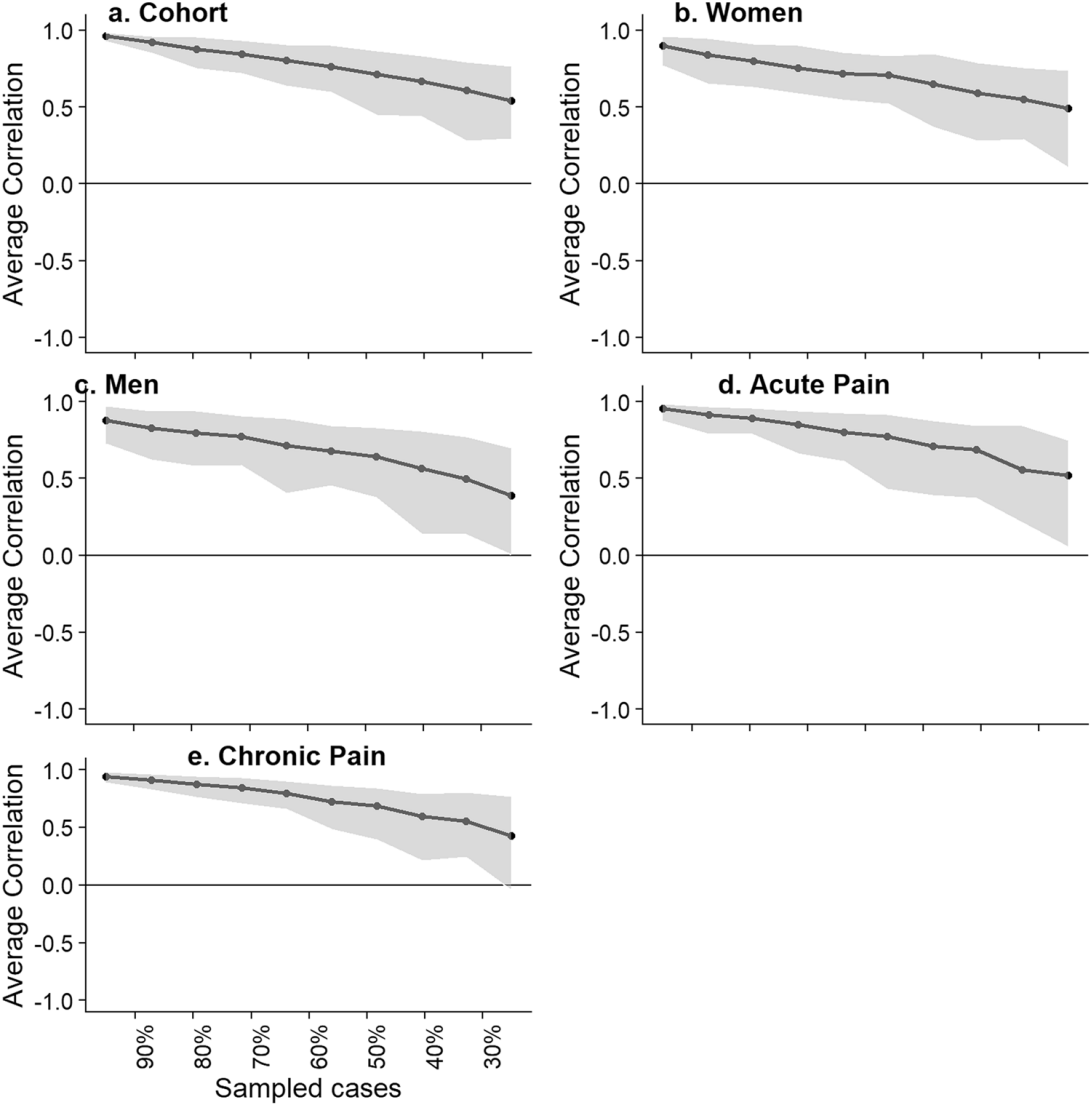
Average correlations between centrality indices. Average correlations between centrality indices of networks sampled with persons dropped and networks built on the entire input dataset, at all follow-up time points. Lines indicate the means and areas indicate the range from the 2.5th quantile to the 97.5th quantile.

The stability (CS_cor_ = _0.7_) of the Strength centrality measure was 0.28, 0.05, 0.05, 0.28, and 0.36 for the cohort, women-subgroup, men-subgroup, acute-subgroup, and chronic-subgroup analyses, respectively (Fig. 7). Considering the low stability of the gender subgroup analyses, these specific results will not be discussed.

## Discussion

This is the first network analysis examining the interplay between pain-related outcomes and biopsychosocial factors during a stringent lockdown, where patients had no access to regular care. The network structures showed important differences in the degree of connectivity, edges (pair-wise interactions), and nodes (variables) with the highest strength, when comparing patients reporting acute versus chronic pain symptoms. Interestingly, the greatest pair-wise associations were observed between pain type (chronic or acute) and pain evolution during lockdown: patients with acute pain had higher chances of reporting worsening during home confinement. Participants’ age, self-reported evolution of pain symptoms, and generalized anxiety symptoms were the variables with the strongest influence on the whole network.

A worsening of chronic pain symptoms linked to the first wave of the COVID-19 pandemic and its associated restrictions has been consistently reported^21,26–29^. Fewer data are available concerning acute pain presentations. Findings from the present study suggest that individuals with acute pain were more likely to report a worsening of their symptoms during lockdown. This is in line with a study showed significantly higher point prevalence in back pain and increased pain intensity during lockdown^72^. Restrictions in the access^25^ and fear of exposure to COVID-19 leading to sharp reductions in visits to the emergency departments^73,74^ may help explain this phenomenon. In contrast, people with chronic pain may have been more prepared for self-managing their symptoms during lockdown^75^. Experts have advocated for diligent adaptations in the management of chronic pain during the pandemic^31,76,77^. However, it could be argued that remote^32^ and in-person^25^ care should also be readily accessible for people with acute pain.

Two of the strongest connections were observed between sadness-worry and anger-helplessness. The number of Google searches for the words sadness and worry increased significantly in countries that introduced full lockdowns in early 2020^78^. These symptoms are generally linked to depression and anxiety respectively, whose prevalence and burden raised during the pandemic^9^. Previous network analyses on data obtained from people with pain highlighted important nodes connecting depression and anxiety^40,79,80^. Surprisingly, although the constructs of worry and anxiety have an intricate relationship^81^, COVID-related worry was associated with reduced generalized anxiety. Worry may play a different role, as suggested by findings that it could mediate the relationship between intolerance of uncertainty and fear of COVID-19^82^. Other state characteristics with a strong bidirectional relationship were anger and helplessness. Cross-sectional studies showed that around 50% of participants were feeling helpless due to the pandemic^83,84^. On the other hand, anger was one of the most common emotional responses to the onset of the pandemic^85^. Both emotions were found to be associated to a lack of information^85,86^ or misinformation^87^, which could be the drivers of this strong connection.

The associations sadness-worry and anger-helplessness inform about important interactions between emotions evoked by the pandemic, however, none of these variables seem to directly interact with the patients’ pain symptoms. Accordingly, pain-related worry experienced by people with chronic pain may have larger impact than other non-pain-related worries^88^. This raises the question regarding whether COVID-related worry has any link to clinical outcomes in people with pain. Although they may be perceived as triggers for pain episodes^26^, there is insufficient evidence supporting a direct link between emotions triggered by the pandemic and pain outcomes^89,90^. This is consistent with our analysis, which shows pain and COVID variables at opposite ends of the network, without strong direct associations. Nonetheless, abundant evidence highlights a bidirectional flow of interactions between pain, emotions, and symptoms of affective disorder^91^, including studies using network analyses^40,79^. Available data support a bidirectional relationship between state anger and chronic pain^92^. Both anger and anger expression were found to influence sensitivity to experimental and clinical pain^93,94^, in a similar way to sadness^95,96^. An indirect effect cannot be ruled out, as was proposed by different models^79,97^. In the present study, the relatively weaker edges linking pain interference to worry and helplessness represent the closest connections between these emotional COVID-related variables and pain outcomes.

Among the variables with the highest strength value, only GAD scores emerged as a potential therapeutic target to impact the largest number of variables. The prevalence of symptoms and diagnoses of anxiety disorders increased dramatically during the pandemic^9^. More than a quarter of the general population in Spain^7,8^ and elsewhere^98^ experienced higher anxiety than pre-pandemic. More severe symptoms and a higher prevalence of anxiety disorders were observed in the youngest^7,98^, which is consistent with our data showing that the younger the individual, the higher the levels of anxiety. These findings suggest that improving access to mental health services should be considered as an important strategy to improve outcomes for people living with pain during a pandemic.

Other variables strongly associated with GAD were predominantly linked to the pandemic, such as COVID-specific anxiety, or stress due to COVID-19, which may have partially driven the augmented symptoms of anxiety^9,98^. GAD was also positively associated with intolerance of uncertainty, while being negatively correlated with self-efficacy. This is not surprising, as the construct of intolerance of uncertainty is used to explain symptoms of worry that are a hallmark of GAD^81^. Intolerance of uncertainty may explain increased levels of anxiety in the face of the pandemic and pandemic-related fear^58,99^. Pain catastrophizing was also strongly associated to GAD, an interaction previously reported in a chronic pain population^80^. This is consistent with data suggesting that anxiety may be a modifiable risk factor influencing pain catastrophizing to reduce pain symptoms^100,101^. Further, catastrophizing had associations with all pain-related variables, and these connections were more abundant in patients with chronic pain. Overall, these findings support the hypothesis that catastrophizing may be pivotal in mediating changes in chronic pain outcomes during COVID-19^21,25,29^.

The subgroup analyses provided reliable data to compare the network structures obtained from people with acute and chronic pain, which showed remarkable differences in connectivity. For those with chronic pain, the network exhibited a higher number of nodes, with greater node strength compared to the network for people with acute pain, suggesting that chronic pain interacts with a larger number of biopsychosocial variables, which should come as no surprise^102^. For patients with acute pain, the sadness–worry pair had greater strength, while for patients with chronic pain, the interaction anger–helplessness had a higher level of significance, which may be related to the strong links shown between state anger and chronic pain^92^.

The connections between pain intensity and interference were strong in both patients’ subgroups, though slightly stronger in the acute pain population. This relationship is likely not a direct one. Evidence suggests that catastrophizing may act as a mediator in patients with acute pain^103^, and during the COVID-19 pandemic^25^. Indeed, catastrophizing interacted with both pain intensity and interference. Additionally, catastrophizing and kinesiophobia showed strong connections, particularly for those with chronic pain, as was previously reported^80^. For acute pain, COVID-related worry and generalized anxiety were identified as the most important nodes, hence are potential therapeutic targets. Interestingly, the three strongest nodes identified in those with chronic pain are not modifiable by clinical intervention (age, gender, marital status). Gender was an important factor in the chronic pain population, while no meaningful associations were seen in the acute pain subgroup. Accordingly, abundant data suggest that women may be more susceptible to chronic pain^104,105^. Although demographics are non-modifiable factors, our data indicate that patient’s age, gender and marital status should be taken into consideration when designing interventions.

## Methodological considerations

The main strength of the present study lies in the novelty and robustness of the statistical analysis. Network analysis provides an integrative approach to the examination of the complex associations between biopsychosocial factors^106^, making of it an excellent tool for the study of people suffering from pain, particularly when chronic. There are, however, limitations to consider. First, being a cross-sectional study, it is not possible to draw any causal or directional inferences of the associations observed. Thus, the interpretation of the results relies on previously published data or models. Considering the exceptional circumstances surrounding the present study, it was deemed preferable to have a data-driven approach to the data rather than the analysis be driven by specific hypotheses. Additionally, two of the questionnaires used had not been validated into Spanish. The FIVE scale, which was published few weeks before the design of this study, was directly translated into Spanish^62^ and a validated version of the IUS–12 was not available in Spanish language, however the original questionnaire with 27 items was^107^. Thus, the items included in the IUS–12 were retrieved from this longer version. Finally, using self-reported measures online may jeopardize the interpretation of the results, although this is common practice in pain research, particularly during COVID-19 social distancing.

## Conclusion

The current study provides evidence for indirect interactions between biopsychosocial factors and pain outcomes obtained during a COVID-19 lockdown. General anxiety emerged as the variable with the strongest connections within the network, and therefore appears to be the preferred potential therapeutic target. Negative emotions evoked by the pandemic such as worry, anger, or anxiety may all indirectly impact pain outcomes, possibly via interactions with pain catastrophizing. Overall, these interactions were stronger in those with chronic pain, for which gender emerged as a key factor. These findings are essential to identify which factors may explain the deleterious effects of both the pandemic and the restrictions on patients living with pain. A better understanding of these interactions will help prioritize care strategies and resources for patients during healthcare emergencies. Overall, improving the access to mental health services for patients with pain may provide an effective approach to reduce the burden of pain conditions during a pandemic.

## Supplementary Information

Supplementary Information.

## Data Availability

The datasets generated during and/or analysed during the current study are available from the corresponding author on reasonable request.
